# Developmental dynamics is revealed in the early Cambrian arthropod *Chuandianella ovata*

**DOI:** 10.1016/j.isci.2021.103591

**Published:** 2021-12-09

**Authors:** Cong Liu, Dongjing Fu, Xingliang Zhang

**Affiliations:** 1 State Key Laboratory of the Continental Dynamics, Shaanxi Key Laboratory of Early Life and Environments, Department of Geology, Northwest University, Xi'an 710069, China; 2 Nanjing Institute of Geology and Paleontology, Chinese Academy of Sciences, Nanjing 210008, China

**Keywords:** Biological sciences, Evolutionary biology, Developmental biology

## Abstract

Segmentation and tagmatization have contributed to the preeminent success of arthropods since their first appearance in the Cambrian. However, the exact mechanism of segmentogenesis is still insufficiently known in living and extinct groups. Here, we describe the postembryonic development of a Waptiid arthropod *Chuandianella ovata* from the early Cambrian Chengjiang biota, South China. The new data illuminate a complex dynamic pattern of anamorphosis and epimorphosis, and a three-step process of segmentogenesis, i.e., the elongation of the terminal segment, delineation of an incipient segment, and full separation of a new segment. Compensatory growth is accomplished by rapid growth of new segments and/or generation of additional segments, which results in the trimorphism of the posterior tagma. Such complex developmental dynamics has rarely been known in the arthropod fossil record and its presence in early history helps to understand the rapid diversification of arthropods in the early Cambrian.

## Highlights

• Dynamic postembryonic growth is recognized in the early Cambrian arthropod *C. ovata*
• *C. ovata* exhibits a three-step mechanism of segmentogenesis
• Adults of *C. ovata* are trimorphic in the segment number of the posterior tagma
• C. *ovata* provides additional evidence for compensatory growth in early arthropods

## Graphical abstract

**Figure:**
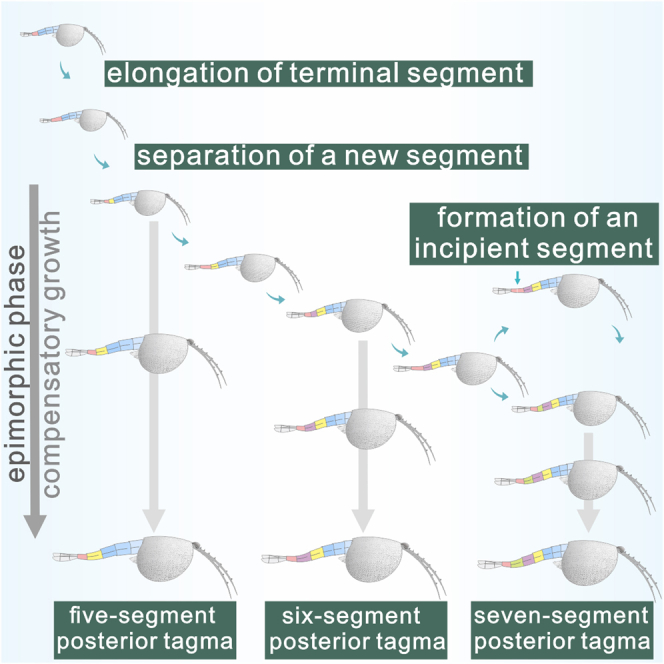

## Introduction

Arthropods are one of the paradigms of segmented animals (Brusca et al., 2016). Regionalization and variation of segments in size, shape, and number are not only responsible for the great success in interspecific diversity and ecology, but also lead to intraspecific polymorphism in many arthropods (Bonato et al., 2003; Carter, 1976; Enghoff et al., 1993; Fu et al., 2014; Fusco and Minelli, 2013; Harrison, 1979, 1980; Liu et al., 2016; Mochida, 1973; Simaiakis, 2009; Zhang et al., 2007; Zhao et al., 2003). In arthropods, segmentation can be completed during the embryonic stages or continues in the post-embryonic development (Clark et al., 2019; Davis and Patel, 2003; Fusco, 2005; Minelli and Fusco, 2004, 2013; Peel, 2004; Peel et al., 2005). Addition of segments during the post-embryonic phase has been recognized across a range of extant and fossil lineages and thus is an important mode of polymorphism in arthropods (Dai and Zhang, 2013; Dai et al., 2016, 2017; Fu et al., 2018; Fusco, 2005; Fusco et al., 2004, 2012; Minelli and Fusco, 2013; Peel, 2004).

In living crustaceans, the mechanism of postembryonic segment formation has been permitted among the conchostracan *Limnadia stanleyana* and the anostracan *Artemia salina*, for which trunk segments are added progressively from the “proliferative zone” lying in front of the telson (Anderson, 1965, 1966). However, in the fossil record, most knowledge on arthropod development comes from the trilobites (Dai and Zhang, 2013; Dai et al., 2016, 2017; Fusco et al., 2004, 2012; Hughes, 2007; Hughes et al., 2017) and phosphatized Orsten-type fossils (Haug et al., 2009, 2010; Müller and Walossek, 1988; Olesen, 2007; Stein et al., 2005, 2008; Walossek, 1993; Walossek and Müller, 1998). Nevertheless, segment formation is rarely captured because of the gap of fossil materials. Except for *Rehbachiella kinnekullensis* (Walossek, 1993) from the Orsten fauna, few fossil taxa demonstrate the process of segment addition though ontogenetic sequences have been established, especially in many trilobites (Dai and Zhang, 2013; Dai et al., 2016, 2017; Fusco et al., 2004, 2012; Hughes, 2007; Hughes et al., 2017; Shen et al., 2014; Zhang and Clarkson, 2009). Our investigation of *Chuandianella ovata* allows a reconstruction of its postembryonic development and recognition of the trimorphic posterior tagma varying from five to seven in the number of segments. Our material also provides the evidence for the segment genesis and compensatory growth of this species.

## Results

### Intraspecific variation of the posterior tagma

**Figure 1:**
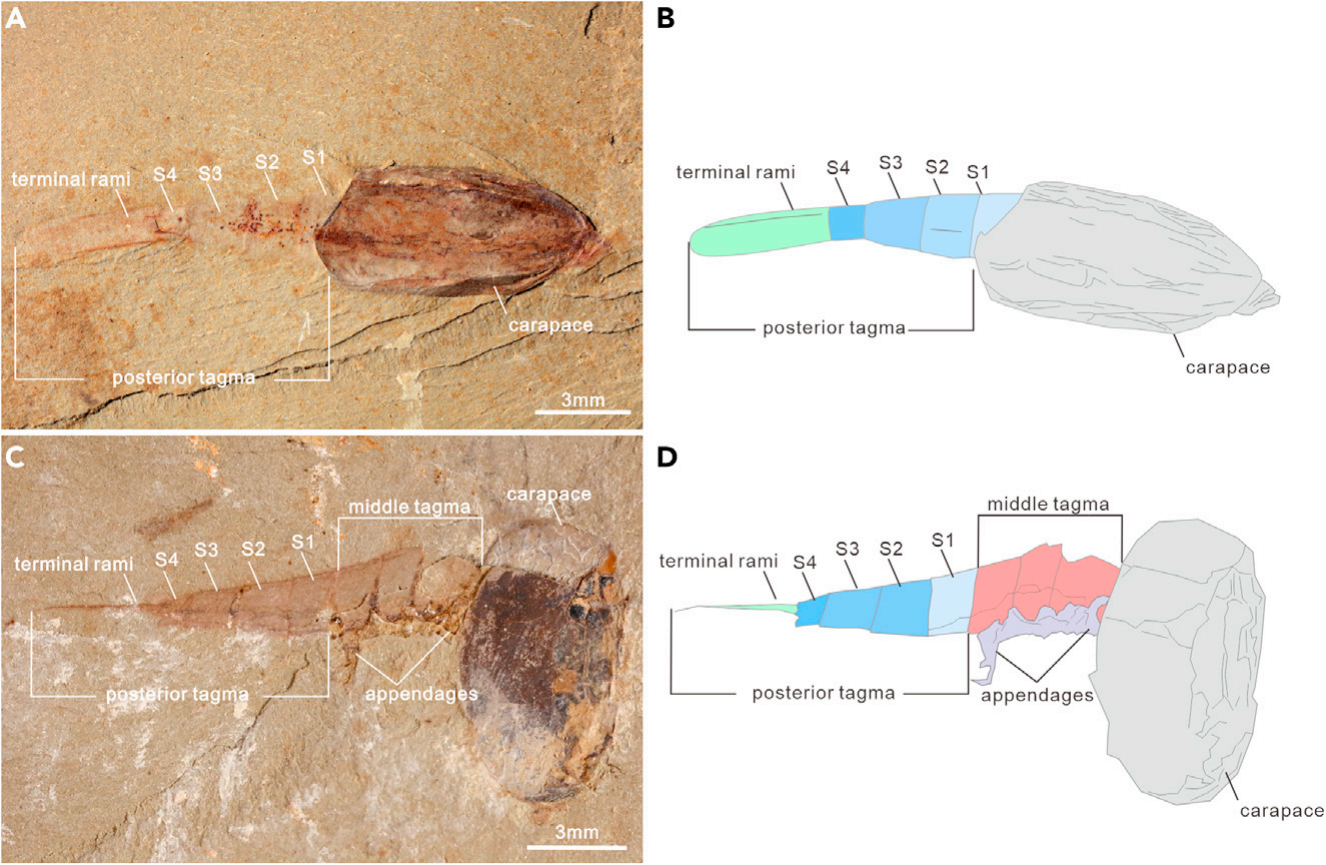
*Chuandianella ovata* with four segments in the posterior tagma (A and B) ELI EJ-506A, immature specimen with four segments (blue area), and a pair of terminal rami (green area) in the posterior tagma; (C and D) ELI JS-183, specimen with dislocated carapace (gray area), illustrating appendages (purple area) associated with the middle tagma (pink area), and four segments in the posterior tagma (blue area). Abbreviations are as follows: S 1-4: first to fourth segments in the posterior tagma.

The waptiid arthropod *C. ovata* is one of the iconic arthropods from the Chengjiang biota (ca. 518 million years before the present) (Hou et al., 2017). The anterior and middle tagmata are covered with a carapace, whereas the posterior tagma protrudes out the posterior end of the carapace (Figures S1 and S2) (Liu and Shu, 2004, 2008; Hou et al., 2008, 2010; Ou et al., 2020; Zhai et al., 2021). However, the middle tagma may be exposed when the carapace is dislocated from the body during the taphonomic process (Figures 1C and 1D). There are 198 complete specimens showing the complete morphology of the posterior tagma varying from 4 to 7 in the number of segments. For the sake of description, we refer to the first segment in the posterior tagma as “S 1” and sort it gradually backwards.

Two small specimens show three limbless segments and a terminal segment with a pair of sub-elliptic terminal rami in the posterior tagma (Figure 1). The length of each segment in the two specimens are measured as: S 1, 0.829 mm and 0.89 mm; S2, 1.055 mm and 0.997 mm; S3, 1.337 mm and 1.463 mm; S4, 1.623 mm and 0.771 mm (Table S1). The total length of the posterior tagma is approximately 3.7mm and 5.1 mm, and shorter than most specimens (Figure 5A). Accordingly, they most probably represent immature specimens.

**Figure 2:**
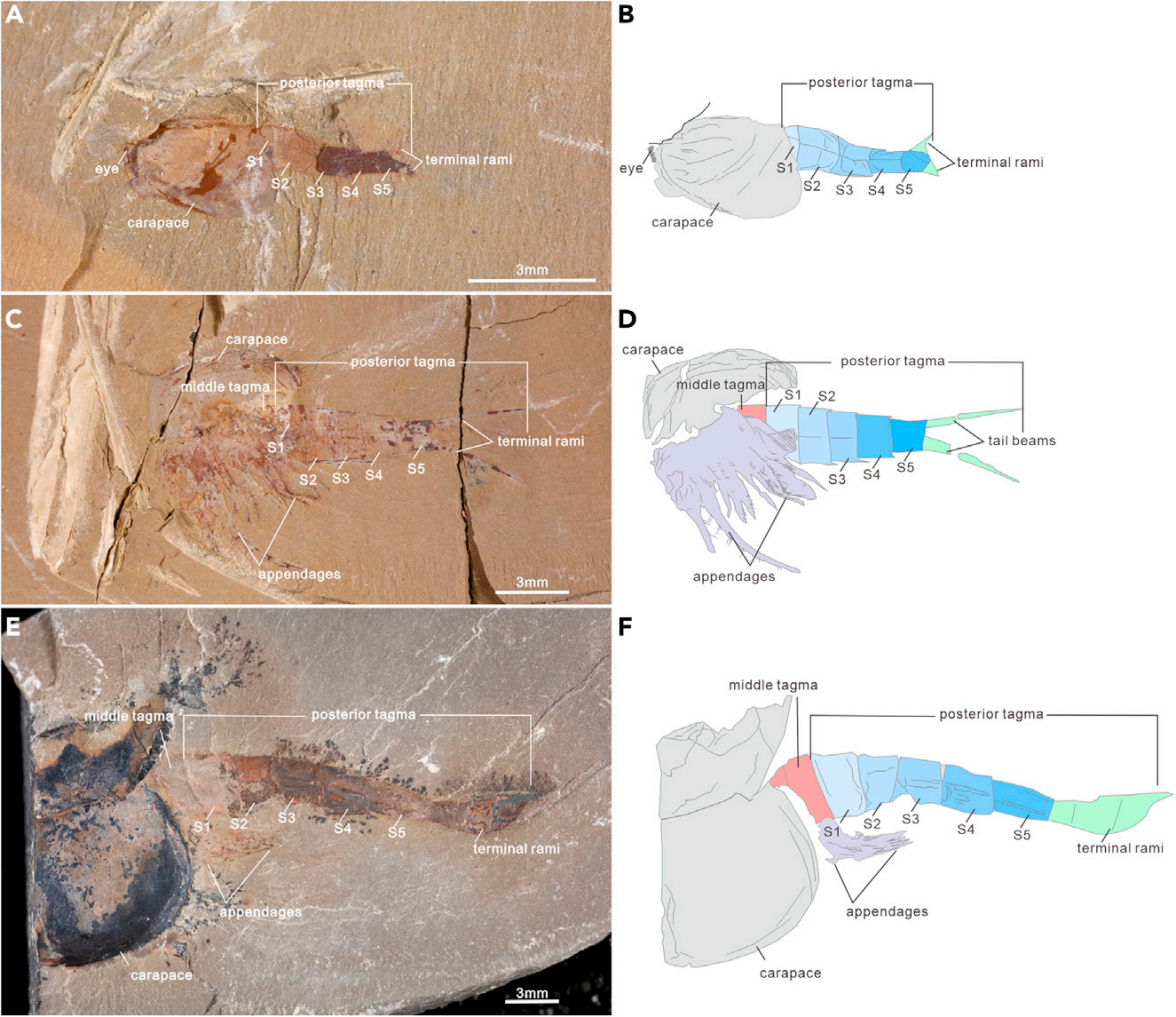
*Chuandianella ovata* with five segments in the posterior tagma (A and B) ELI SJZ-B19-657, small specimen with five segments (blue area), and fragment terminal ramus (green area); (C and D) ELI JS-655, posterior tagma carrying five segments (blue area) armed with posteriorly directed spines, and segments in the middle tagma (pink area) bearing appendages (purple area); (E and F) ELI SJZ-B23, largest specimen in our collection consisting of a five-segment posterior tagma. Abbreviations are as follows: S 1-5: first to fifth segments in the posterior tagma.

Fifteen specimens carry five segments in the posterior tagma, ranging from 2.898 to 13.055 mm in length (Figures 2 and 5A). In the posterior tagma, the first three segments become elongated toward the rear, whereas the length of the last two segments is variable in specimens. Four specimens are characterized by the S 3 being the longest and the length of S 4 and S 5 increasingly decreased (Table S1). In nine specimens the length of S 4 is the longest, significantly longer than S 5, and the length ratios of S 5 to S 4 (LS 5/LS 4) range from 0.48 to 0.95. Additional two specimens were featured by S 5 being the longest (Figures 6A and 6B), and therefore segments in the posterior tagma become increasingly elongated. The S 5/S 4 length ratio values are 1.20 and 1.29, respectively.

**Figure 3:**
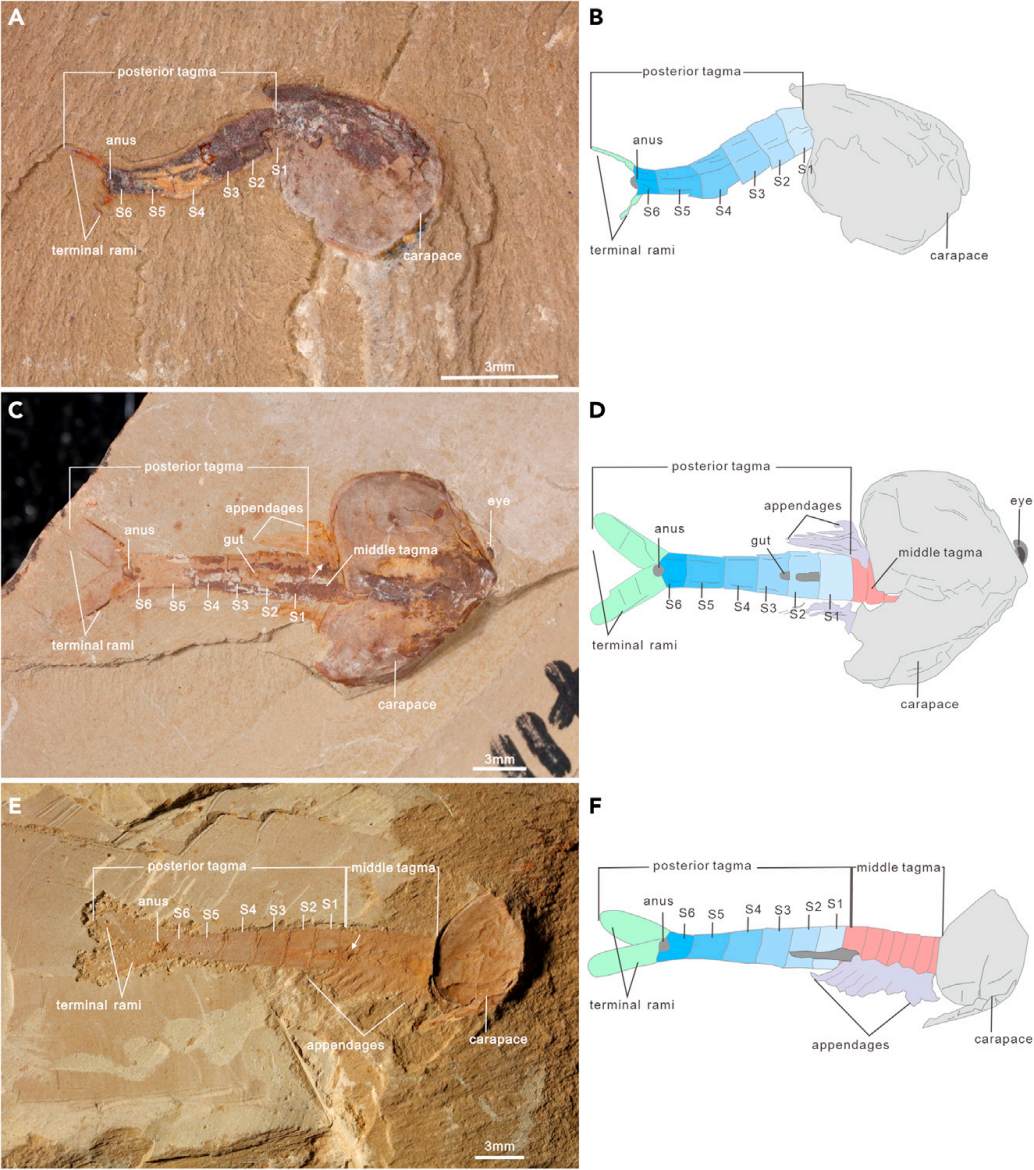
*Chuandianella ovata* with six segments in the posterior tagma (A and B) ELI MF-716A, small specimen with six segments (blue area) in the posterior tagma, and an anus on the posterior edge of the terminal segment; (C and D) ELI EJ-411A, specimen with six segments (blue area), and a pair of terminal rami (green area) in the posterior tagma; (E and F) ELI JS-031B, larger specimen with six segments (blue area) in the posterior tagma. The middle tagma is composed of isometric segments and appendages (purple area). The last appendage is indicated by an arrow. Abbreviations are as follows: S 1-6: first to sixth segments in the posterior tagma.

Specimens with six segments in the posterior tagma are dominated in number. A total of 144 specimens have been recognized, accounting for 72% of specimens with a complete posterior tagma (Figure 3). The length of the posterior tagma ranges from 2.82 to 12.229 mm (Figure 5A). Similar to specimens with a five-segment posterior tagma, this largest group can be further subdivided into three categories according to the position of the longest segment. Two specimens with S 4 being the longest, in which the length of S 1 to S 4 gradually increases, whereas the length of the last two segments gradually decrease toward the rear. In these two specimens, S 5 is just slightly shorter than S 4, with the length ratio about 0.99. There are 138 specimens with S 1-5 becoming increasingly longer posteriorly. In these species, S 5 is the longest, and S 6 is consistently shorter than S 5. The length ratios of S 6/S 5 vary from 0.31 to 0.89. In addition, four specimens have a much longer S 6 (Figures 6C and 6D), the length ratios of S 6/S 5 are 1.15, 1.32, 1.48, and 1.60, respectively (Table S1). Surprisingly, a superficial intersegmental groove is present in the longest terminal segment of ELI JS-027 (Figures 6C and 6D), probably representing a prelude to separate a new segment.

**Figure 4:**
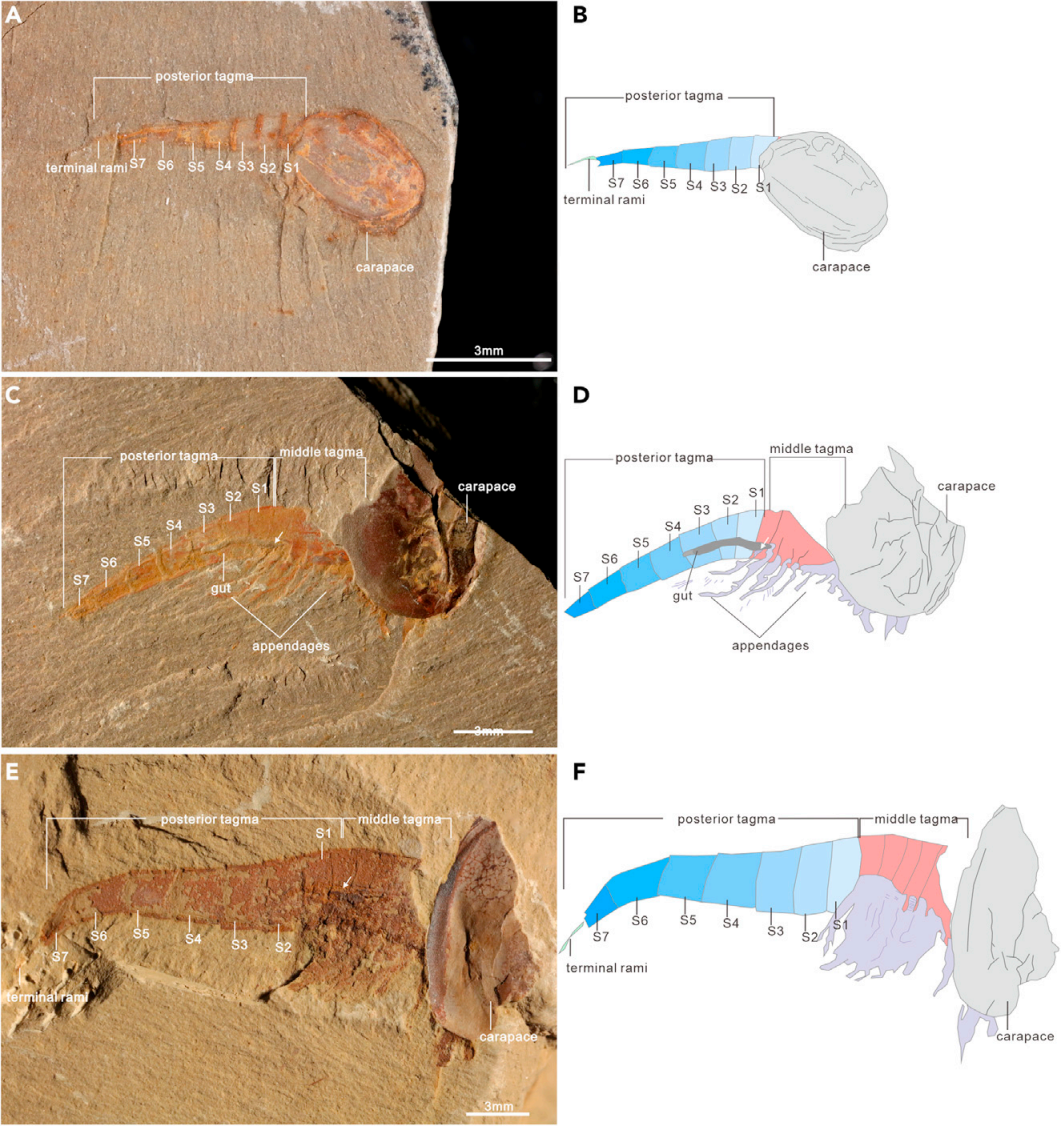
*Chuandianella ovata* with seven segments in the posterior tagma (A and B) ELI JS-397A, small specimen with a seven-segment (blue area) posterior tagma; (C and D) ELI SJZ-B14-741A, specimen with seven segments (blue area) in the posterior tagma. The middle tagma (pink area) contains isometric segments, gut (dark gray area), and appendages (purple area). The last appendage is indicated by an arrow; (E and F) ELI JS-233, larger specimen with seven segments (blue area) in the posterior tagma. The last appendage is indicated by an arrow. Abbreviations are as follows: S 1-7: first to seventh segments in the posterior tagma.

**Figure 5:**
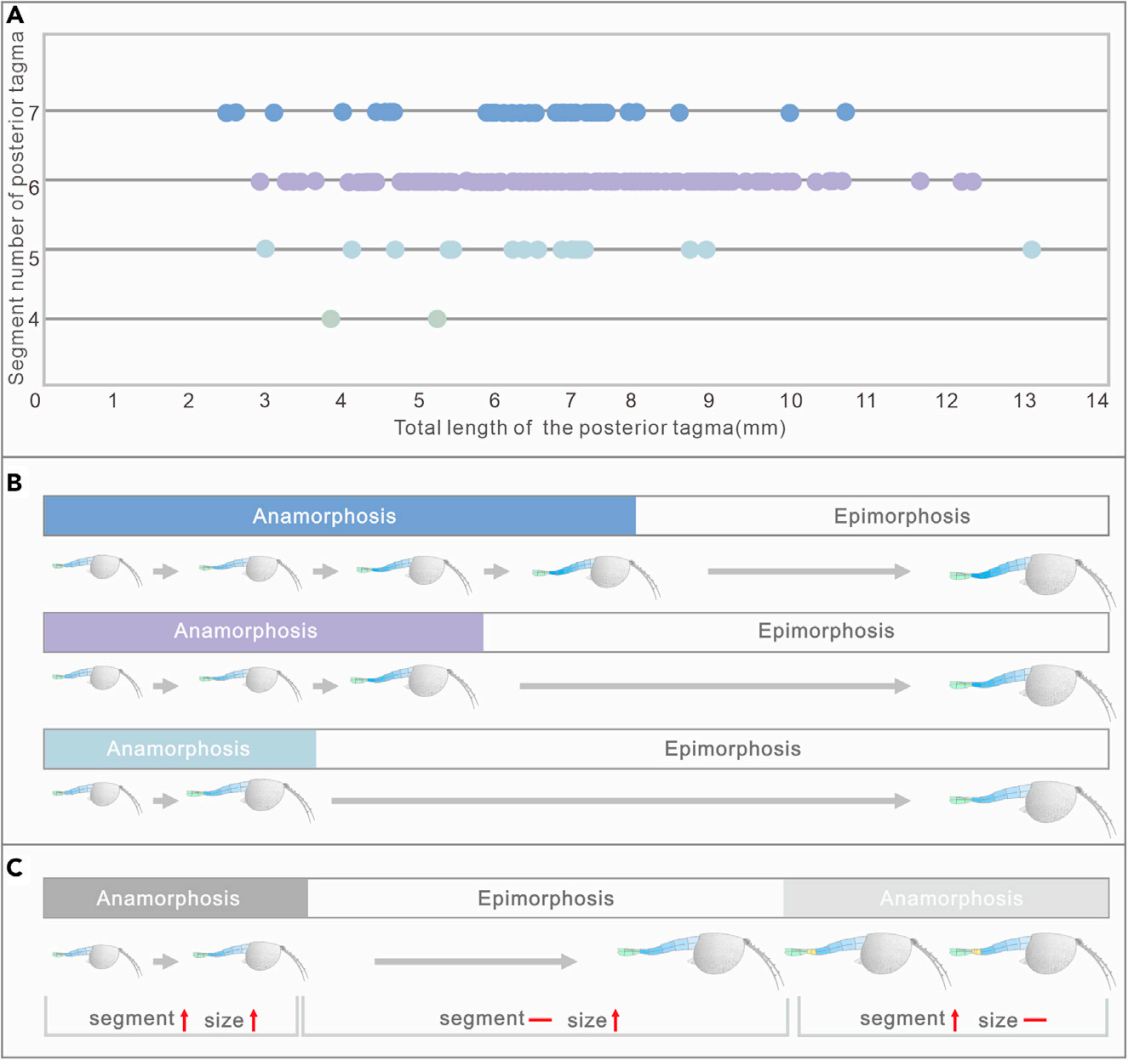
Statistical data and postembryonic ontogenetic pattern of *Chuandianella ovata* (A) statistical measurements showing variations of the segment number and the total length of the posterior tagma (Table S1. Data of segment length in the posterior tagma of *Chuandianella ovata*). Four-segment specimens (n = 2); five-segment specimens (n = 15); six-segment specimens (n = 144); seven-segment specimens (n = 35); (B) dynamic post-embryonic development of the trimorphic posterior tagma in *C. ovata*; (C) a less likely model of postembryonic development of *C. ovata*. Segment —: invariant in segment number of the posterior tagma; segment ↑: increase in segment number of posterior tagma; size —: invariant in individual size; size ↑: increase in individual size.

**Figure 6:**
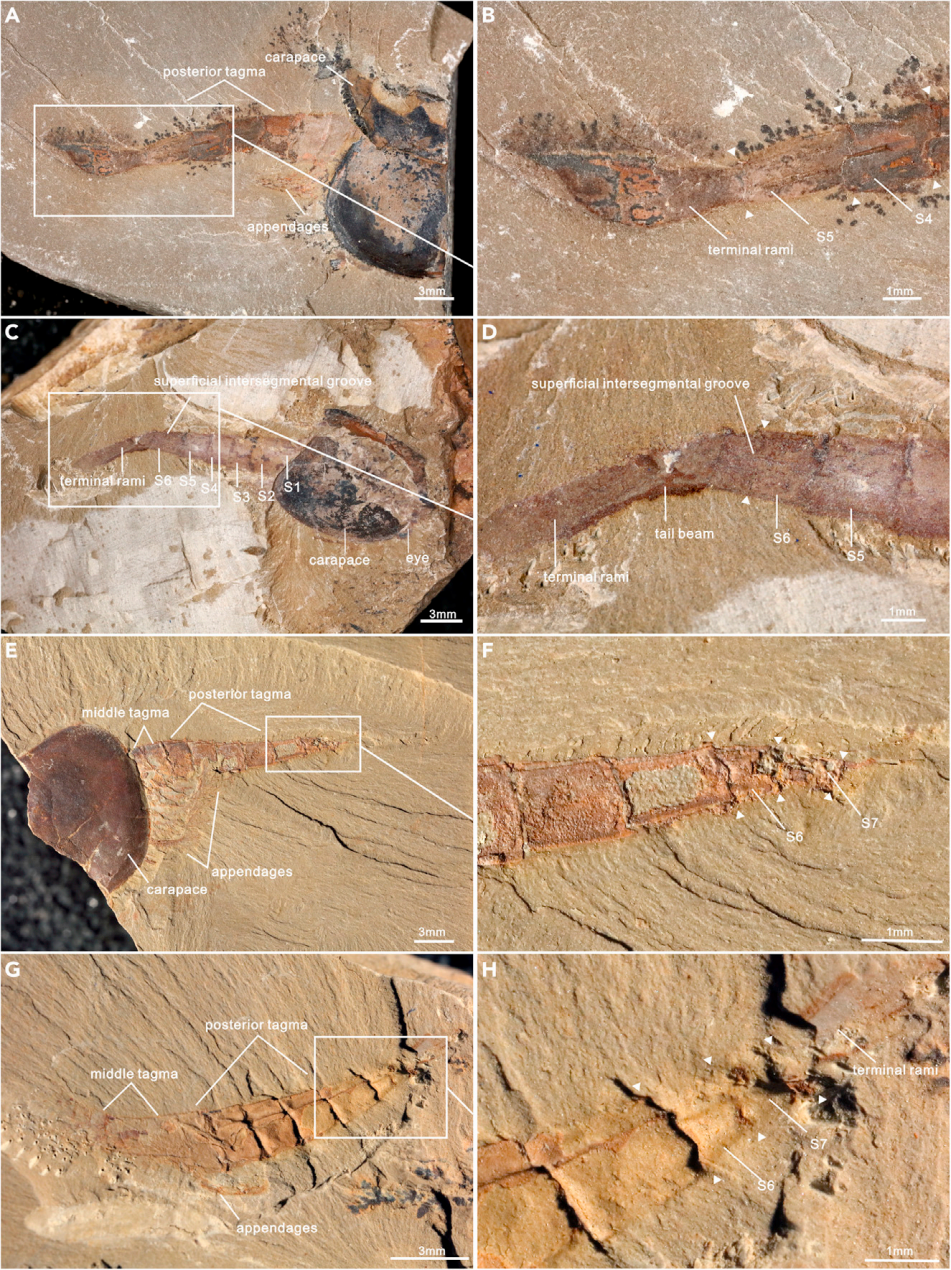
The process of the segment genesis in *Chuandianella ovata* (A and B) ELI SJZ-B23, specimen with five segments in the posterior tagma; (B) magnification of the rear of the posterior tagma, showing the elongated terminal segment (S 5). The triangles indicate the division of the segment; (C and D) ELI JS-027, specimen with a six-segment posterior tagma; (D) magnification of the lengthened terminal segment, showing a superficial intersegmental groove (indicated by triangles) at the mid length of the segment; (E and F) ELI SJZ-B20-900A and (G and H) ELI JS-047, specimens with seven segments in the posterior tagma; (F and H) magnification of the rear of posterior tagma, showing a significantly shorter S 6. The triangles indicate the division of the segment. Abbreviations are as follows: S 4-7, fourth to seventh segments in the posterior tagma.

There are 37 specimens with seven segments in the posterior tagma (Figure 4), ranging in length from 2.379 to 10.608 mm (Figure 5A). Two groups can be identified according to the length of segments. In 33 specimens, the length of S 1-6 is increasing gradually. S 6 is the longest, and S 7 is shorter than S 6. In the remaining four specimens, S 1-5 gradually increases in length, S 5 reaches a maximal length, and thereafter the last two segments decrease in length. There are two specimens with S 6 significantly shorter than S 5; the length ratios of LS 6/LS 5 are 0.39 and 0.41, respectively (Figures 6E–6H). Therefore, these short S 6 likely represent newly generated segments.

### Statistical analysis of the length of posterior tagma

To determine the differences among four categories of specimens with different numbers of segments in the posterior tagma, 198 specimens were used for principal component analysis (PCA). The result shows no variations among the four groups (Figure S3B), indicating that they are identical in the length range of each segment in the posterior tagma. Among the four categories, the total length of the posterior tagma and each length of the last four segments are referred as independent (X) and dependent (Y) variables, respectively. The regression analysis shows: Line for the fourth segment to last: Y = 0.19296X + 0.060158, R^2^ = 0.93246; Line for the antepenultimate segment: Y = 0.20724X + 0.077246, R^2^ = 0.93559; Line for the penultimate segment: Y = 0.23228X + 0.10558, R^2^ = 0.85075; Line for terminal segment: Y = 0.14821X + 0.092442, R^2^ = 0.49731 (Figure S3A). R^2^ represents the coefficient of determination. The statistical analysis shows that the terminal segment has high dispersion, whereas the other three segments have good linear fitting, and the slope increases gradually from the reciprocal fourth to second segment. Thus, the length of the terminal segment has poor stability, and the rest three segments are gradually lengthening posteriorly. In addition, in the violin diagram (Figure S3C) the length range of the terminal segment reveals a swelling ranging from 3 to 3.5mm in specimens with a five-segment posterior tagma, which are not present in the six- or seven-segment specimens. Hence, the length of the terminal segment has a wide range of variation, especially in specimens with a five-segment posterior tagma. Meanwhile, the vioplot also indicates that both the median and mean values of the penultimate segment are larger than those of the terminal segment among all four categories of specimens (Figure S3C).

## Discussion

### Anamorphosis and trimorphism

**Figure 7:**
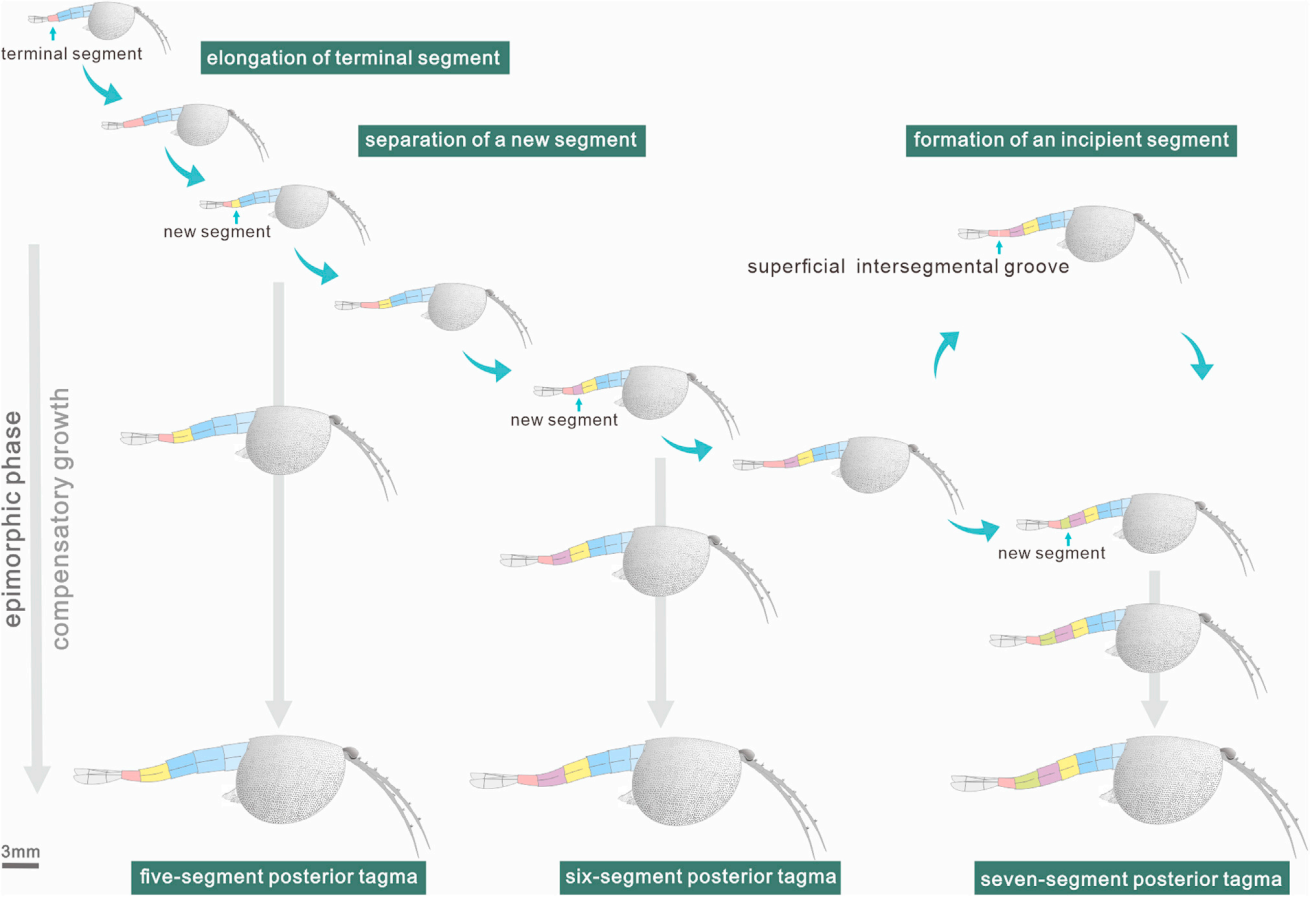
Reconstruction of the dynamic post-embryonic development of *Chuandianella ovata*

Morphological analyses reveal four phenotypes varying from four to seven in the segment number of the posterior tagma (Figures 1, 2, 3 and 4). The consistent morphological characteristics of the four phenotypes indicate that *C. ovata* does not possess metamorphic development, but follows an anamorphic developmental mode because of segment addition during the postembryonic ontogeny. The phenotype with a four-segment posterior tagma is small, represented by only two specimens, whereas the sizes of these two specimens are both larger than the smallest individual among phenotypes with five-segments (Figures 1 and 5A). Hence, these four-segment specimens can be interpreted as immaturities of the five-segment phenotype. Each of the other three phenotypes are represented by numerous specimens (Table S1). Such interspecific variation in the number of segments were usually considered as a series of growth stages of a single phenotype, e.g., *Fuxianhuia protensa* (Fu et al., 2018), *Rehbachiella kinnekullensis* (Walossek, 1993), and many trilobites (Dai and Zhang, 2013; Dai et al., 2016, 2017; Fusco et al., 2004, 2012). However, this is less likely the case of *C. ovata*. First, measurements demonstrate that the posterior tagmata of these five-segment, six-segment, and seven-segment phenotypes have a similar size range from immaturities to adults, approximately 3 to 13 mm long (Figure 5A). Moreover, principal component analysis indicated that the four categories of specimens could not be distinguished according to the length of each segment in the posterior segment (Figure S4B). Third, the largest specimen (ELI SJZ-B23) carries a five-segment posterior tagma (Figures 2E and 2F), whereas the smallest (ELI SJZ-B16-604) bears a seven-segment posterior tagma (Figure 5A; Table S1). The consistent morphology of the four phenotypes indicates that these specimens acquired adult morphology during the postembryonic development. Therefore, it is less likely that the significantly increased five-segment specimens represented “giant larval” as seen in the extant and extinct crustacean larvae (Gundi et al., 2020; Nagler et al., 2017). The size decrease during the postembryonic development is presently unknown among extant and extinct arthropods. In addition, if the three phenotypes represent a successive developmental sequence, the postembryonic development of *C. ovata* would consist of three phases: the anamorphosis with the segment number of the posterior tagma increasing to five, followed by an epimorphosis during which the size increases whereas the segment number of the posterior tagma remains as five, and then anamorphosis again with addition of two more segments (Figure 5C). Such a three-phased pattern of postembryonic development has not been reported in either fossil or living arthropods. Therefore, it is most probably that *C. ovata* has a trimorphic posterior tagma varying from five to seven in the segment number. The postembryonic development of each phenotype involves two phases: anamorphosis with both segment number and size increasing, followed by epimorphosis solely increasing size (Figure 5B and 7).

Intraspecific polymorphism is common among extant arthropods (Fusco and Minelli, 2013; Minelli and Fusco, 2004). In terms of morphology, flight polymorphisms in insects are the most obvious examples, e.g., an orthopteran cricket *Allonemobius fasciatus* and a small dipteran fly *Plastosciara perniciosa*, both of which have macropterous and micropterous phenotypes (Roff, 1984; Steffan, 1974). In addition, because of perturbation of external factors, such as diet, temporary starvation, and low/high temperature (Esperk et al., 2007), the variation in the number of segments is also widespread among extant arthropods (Fusco and Minelli, 2013; Uliana et al., 2007; Pereira et al., 1994; Simaiakis, 2009; Schileyko, 2006; Chagas et al., 2008; Enghoff et al., 1993). For instance, the number of leg-bearing segments of geophilomorph *Mecistocephalus microporus* is calculated from 93 to 101 (Bonato et al., 2003). In the fossil record, intraspecific variation of segment number and morphology had also been recognized in many taxa, such as *Misszhouia longiacaudata* (Zhang et al., 2007), *Isoxys auritus* (Fu et al., 2014), and many trilobites, e.g., *Duyunaspis duyunensis* (Dai et al., 2017). However, the polymorphism was formally reported in a limited number of cases, e.g., the early Cambrian arthropod *Fuxianhuia protensa* (Fu et al., 2018) and the middle Silurian trilobite *Aulacopleura konincki* (Fusco et al., 2004, 2012; Hughes, 2007; Hughes et al., 2017). At adult phase, *F. protensa* bears 26 to 30 trunk segments (Fu et al., 2018), and *A. koninckii* has five morphs with 18 to 22 thoracic segments (Fusco et al., 2004, 2012; Hughes et al., 2017). The recognition of the trimorphic posterior tagma in *C. ovata*, provides an additional example of polymorphism in fossil arthropods.

### Genesis of new segments

In our collection, two seven-segment (ELI JS-047 and ELI SJZ-B20-900A) and one six-segment (ELI JS-027) specimens demonstrate the genesis of new segments in the posterior tagma. In the two seven-segment specimens, the length of S 6 is significantly shorter than S 5 (Figures 6E–6H), which contradicts the general trend of posterior segments elongating toward the rear. Moreover, both regression analysis and vioplot show a broader range in the length of the terminal segment, which are probably attributed to subterminal separation of a new segment. Consequently, the terminal segment was relatively longer upon separation and shorter immediately after separation (Figure S3A and C). In our research, there are six specimens with a much longer terminal segment in the five-segment and six-segment phenotypes (Figures 6A–6D; Table S1). Among them, the terminal segment of the specimen ELI JS-027 shows a shallow groove at the mid length (Figures 6C and 6D), reminiscent of the case in the “Orsten” type crustacean *R. kinnekullensis*, for which the formation of trunk segment is characterized by two steps. Before a new segment finally delineates from the terminal end, there is an intermoult stage with fissure on the dorsal surface (Walossek, 1993). Therefore, it is reasonable to assume that posterior segments of *C. ovata* could be generated through three steps, including the elongation of the terminal segment, the formation of blurred incipient segment (shallow groove) and a separation of new segment in the subterminal region (Figure 7). Such segmentogenesis is basically concordant with living crustaceans *Limnadia stanleyana* and *Artemia salina*, for which post-mandibular segments are progressively proliferated from a growth zone lying in the anterior region of the telson (terminal segment) (Anderson, 1966). The post-protaspid ontogeny and the pattern of segment release from the pygidium have been extensively studied in trilobites, such as *Duyunaspis duyunensis* (Dai et al., 2017), *Eoredlichia intermediate* (Dai et al., 2016), and *Aulacopleura koninckii* (Fusco et al., 2004, 2012; Hughes, 2007; Hughes et al., 2017). However, the exact mechanism of new segment genesis is still poorly understood. The present study provides valuable evidence for the postembryonic segmentogenesis in an early Cambrian arthropod.

### Compensatory growth

Target phenotype refers to a phenotype determined by the individual genetic makeup without perturbing factors (Minelli and Fusco, 2013; Nijhout and Davidowitz, 2003). Therefore, during the postembryonic development of arthropods, there should be a series of target character states at each stage of ontogeny, if external conditions such as temperature, nutrition, and parasitism are consistent (Minelli and Fusco, 2013). However, the majority of arthropods deviate from the target trajectory as a result of variations in the external factors (Fusco and Minelli, 2013; Hartnoll, 1982; Nijhout and Davidowitz, 2003), and hence need to adjust via compensatory growth which is accomplished by altering the number of stages and/or a stage-by-stage feedback mechanism to reach the target features (Minelli and Fusco, 2013).

In our collection of *C. ovata*, more than 91% of the specimens have the longest penultimate segment (Table S1), but the length of newly formed segments is significantly shortened (Figures 6E–6H). The same phenomenon was observed in growth stages of *R. kinnekullensis*, where the incipient segment was shorter (in Walossek, 1993, plate 14; 17; 20; 23) (Walossek, 1993). Therefore, compensatory growth is necessary for newly separated short segments to achieve the target length during anamorphic phase. Compensatory growth is also the mainspring of the trimorphism seen in *C. ovata*. Statistic measurements demonstrate that the six-segment posterior tagma is the dominant phenotype (Figure 5A), accounting for more than 72% of specimens. Accordingly, in the absence of compensation, only the six-segment posterior tagma would be present in the epimorphic phase. However, three phenotypes have been recognized in the adulthood of *C. ovata* (Figure 5A). Specimens with a five-segment posterior tagma started their epimorphic development ahead of schedule because of the excessively rapid elongation rate of new segments, and hence reached their target size one stage earlier (Figure 5B). Although specimens with a seven-segment posterior tagma entered the epimorphic phase one stage later, they grew to the target size through a slower elongation rate of new segments or relative rapid generation of new segments (Figures 5B; S4C). Consequently, these compensatory growth modes are responsible for the trimorphic posterior tagma reaching the target size. Similar compensatory growth was reported in many extant arthropods (Hartnoll and Dalley, 1981; Klingenberg, 1996; Tanaka, 1981; Twombly and Tisch, 2000; West and Costlow, 1987). Typically, in the postembryonic development of the tobacco hawkmoth *Manduca sexta* and the cockroach *Blattella germanica*, individuals reach the target threshold size by increasing the number of developmental stages (Tanaka, 1981; Nijhout, 1975). The dynamics of segment generation and growth seen in *C. ovata* is as complex as extant representatives.

### Conclusion

The waptiid arthropod *Chuandianella ovata* from the 518 million years old Chengjiang biota carries a trimorphic posterior tagma with five to seven segments and demonstrates a complex developmental pattern. Each phenotype underwent an anamorphic phase during which a fixed number of segments in the posterior tagma are obtained, followed by an epimorphic phase without new segment addition when molting (Figure 7). The three phenotypes are comparable in size range, and the one with six segments is dominant in the number of specimens (Figure 5A). *C. ovata* demonstrates a similar compensatory growth mechanism to living arthropods. In particular, during the anamorphic phase, the generation and growth of new segments were coupled dynamically, and hence *C. ovata* reached its target size through increasing or decreasing developmental stages (Figure 5B). As a result, some had a segment growth rate exceeding its generation rate and thus started their epimorphosis relatively earlier at the stage with five segments in the posterior tagma, the majority went the epimorphic stage when the posterior tagma had six segments, whereas others had a relatively later epimorphic stage with one more segment when the segment generation rate exceeds its growth rate. Accordingly, the adults maintain the trimorphism in the posterior tagma (Figure 7). The newly generated segment was short, separated from the subterminal region of the terminal segment, and reached its normal length through an accelerating growth rate. The trimorphism and developmental pattern seen in *C. ovata* are rarely known in fossil records and reveals that the complicated dynamics of segment growth and generation evolved early in arthropod history.

### Limitations of the study

Fossils used in our study are unlikely to represent a single population in the strict sense, which is always the case in paleontological studies. However, in our study, all specimens were collected from a narrow stratigraphical range of the *Eoredlichia*-*Wu*ti*ngaspis* trilobite biozone (Hou et al., 2017; Zhang et al., 2007) and are generally considered to reflect the size and morphological range of “a single population”.

## STAR★Methods

### Key resources table

**Table:** 

REAGENT or RESOURCE	SOURCE	IDENTIFIER
**Software and algorithms**		

CorelDraw X9	Bouton, 2008	https://www.coreldraw.com/
Adobe Photoshop CC	Press, 2010	https://www.adobe.com/
ImageJ 1.8.0	Burger and Burge, 2006	https://imagej.nih.gov/ij/
PAST v3.12	Hammer et al., 2001	https://past.en.lo4d.com
Power BI	This paper	https://powerbi.microsoft.com/zh-cn/

### Resource availability

#### Lead contact

Further information and requests for data should be directed to and will be fulfilled by the lead contact, Xingliang Zhang (xzhang69@nwu.edu.cn).

#### Materials availability

This study did not generate new unique materials.

### Experimental model and subject details

In this study, the research object is fossil, and no experimental models.

### Method details

#### Materials

A total of 1459 specimens from eight localities of the Chengjiang biota, i.e. Chengjiang, Ercai, Erjie, Jianshan, Mafang, Sanjiezi, Shankou and Tanglipo (CJ, EC, EJ, JS, MF, SJZ, SK, TLP) were analyzed in this study. Specimens were collected from the Maotianshan Shale Member (Member 3) of the Yu'anshan Formation (Hou et al., 2017; Zhang et al., 2007). All the studied specimens have been deposited in the Shaanxi Key Laboratory of Early Life and Environments (LELE), Northwest University, Xi'an.

#### Methods

All specimens were observed using stereomicroscopes and photographed by a Canon EOS 5D Mark Ⅱ camera under incandescent lamp. Camera lucida drawings were made using a Nikon SMZ 100 stereomicroscope and prepared with CorelDraw X9 (Bouton, 2008). All images were processed in Adobe Photoshop CC (Press, 2010).

#### Terminology

The morphological and ontogenetic terms are derived from Vannier (2018), Olesen (2013) and Minelli and Fusco (2013) (Minelli and Fusco, 2013; Olesen, 2013; Vannier et al., 2018). Previous interpretation of the morphology contributes to place *C. ovata* within Waptiidae (Pancrustacea) (Hou et al., 2008, 2010, 2017; Liu and Shu, 2004, 2008; Ou et al., 2020; Vannier et al., 2018), thus the technical terms to indicate morphology are derived from *Waptia fieldensis*, i.e., *carapace* (Olesen, 2013; Vannier et al., 2018). The body of *C*. *ovata* can be subdivided into three tagmata: *the anterior tagma*, *middle tagma* and *posterior tagma* (Figure S1). The anterior and middle tagmata bear appendages and are covered with a “bivalved” carapace, while the posterior tagma is extending beyond the carapace (Figures S1 and S2) (Hou et al., 2008, 2010, 2017; Liu and Shu, 2004, 2008; Ou et al., 2020; Zhai et al., 2021). The terminal segment in the posterior tagma is consisted of a pair of sub-elliptic *terminal rami* (Figures S1 and S2). In the text, we use ontogenetic terms recommended in Minelli and Fusco (2013). During the post-embryonic development of arthropods, *segmentation* is consisted two concepts, including the production and differentiation of segments (Minelli and Fusco, 2013). Development by *anamorphosis* is the sequence of changes involved in the evolutionary development, for which is characterized by metamorphosis with post-embryonic increment in segment number (Minelli and Fusco, 2013), while *epimorphosis* is a form of development with no post-embryonic increment in segment number (Minelli and Fusco, 2004, 2013; Fusco, 2005).

### Quantification and statistical analysis

Size measurements were taken from the photographs by the software ImageJ 1.8.0 (Burger and Burge, 2006) (Table S1. Data of segment length in the posterior tagma of *Chuandianella ovata*). Two kinds of values were documented, including the length of each segment and the total length of the posterior tagma. The segments in the posterior tagma are tubular shape, so there is no discrepancy in the length between specimens with dorsal and lateral preservation. For each segment in posterior tagma, the length is the straight distance between the midpoints of the anterior and posterior edges. The total length is the sum of each segment lengths excluded the terminal rami. Regression analysis of the total length of posterior tagma (independent variables (X)) and each length of last four segments (dependent variables (Y)) was performed by PAST v3.12 (Hammer et al., 2001 and Figure S3A). R^2^ represents the coefficient of determination, which reflects the degree of regression model fits the observed data. The vioplot of the penultimate and terminal segments was plotted by and Power BI (Figure S3C).

A total of 198 specimens (two four-segment specimens, 15 five-segment specimens, 144 six-segment specimens and 37 seven-segment specimens) were analysed by principal component analysis (PCA). We utilized with the total length and last four segments length in the posterior tagma among four categories in this analysis. The analysis data has been deposited at Table S2 (Table S2. Data of principal component analysis). Principal component analysis was performed by PAST v3.12 (Figure S3B).

## Data Availability

Data: All fossil materials have been deposited in the Shaanxi Key Laboratory of Early Life and Environments (LELE), Northwest University, Xi'an. Data reported in this paper will be publicly available in supplementary tables as of the date of publication. Code: This paper does generate and report original code. Any additional information required to reanalyze the data reported in this paper is available from the Lead Contact upon request.

## Supplemental information

Document S1. Figures S1–S3 and Table S1 and S2

Table S1. Data of segment length in the posterior tagma of *Chuandianella ovata*, related to Figure 5 and the STAR Methods

Table S2. Data of principal component analysis, related to the STAR Methods
