# Supplementary material for: Developmental dynamics is revealed in the early Cambrian arthropod Chuandianella ovata
Source: iScience. 2021 Dec 9;25(1):103591. doi: 10.1016/j.isci.2021.103591 (PMC8717428; doi:10.1016/j.isci.2021.103591)
Supplement: Document S1. Figures S1–S3 and Table S1 and S2 [file mmc1.pdf]

iScience, Volume *25*

Supplemental information

Developmental dynamics is revealed in the early Cambrian arthropod *Chuandianella ovata*

Cong Liu, Dongjing Fu, and Xingliang Zhang

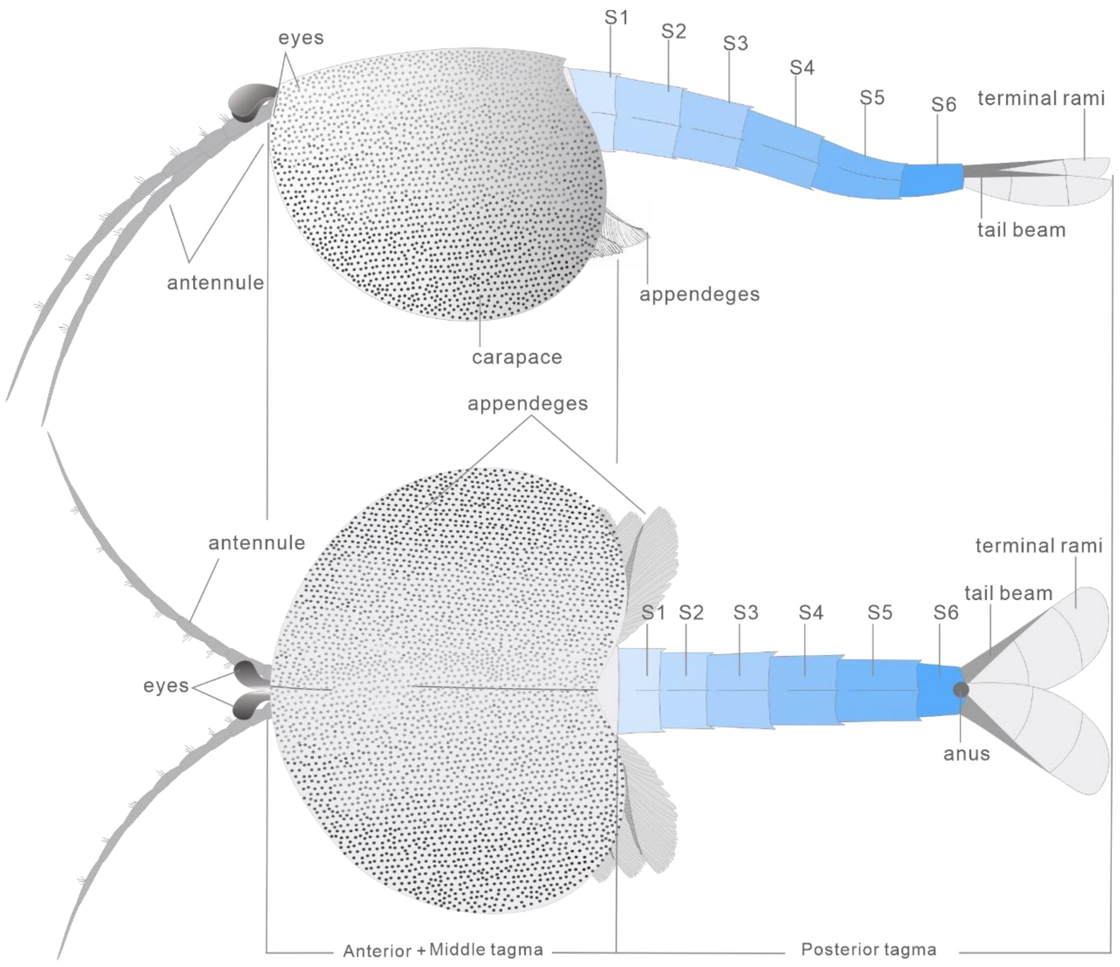

Figure S1. Gross morphology of *Chuandianella ovata*, Related to the STAR METHODS. Upper: lateral view. Lower: dorsal view.

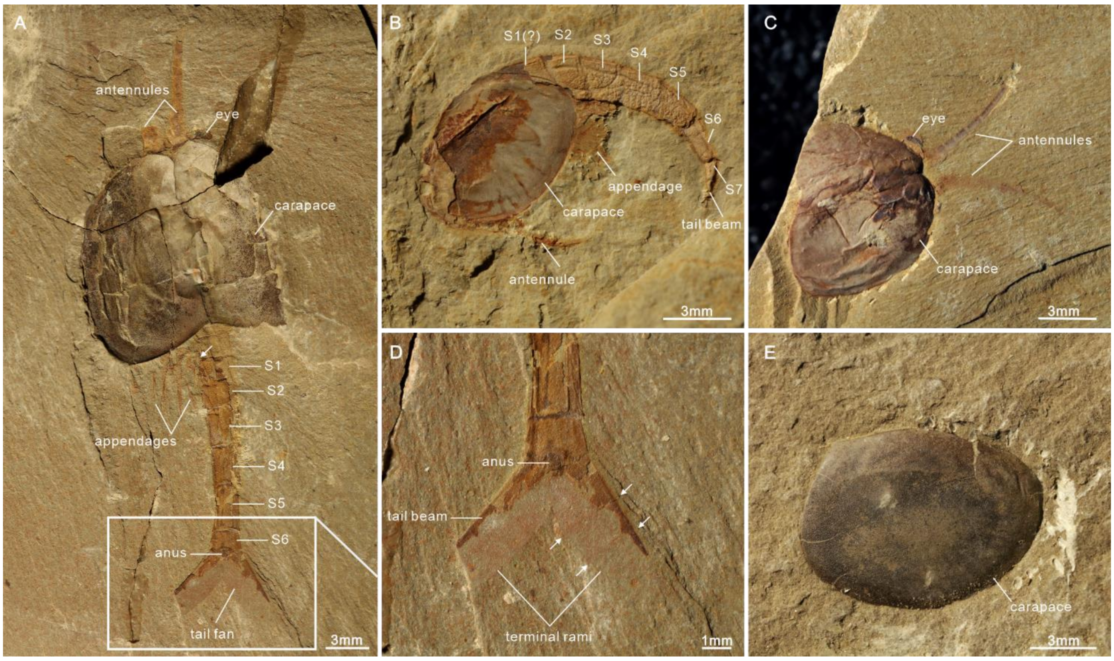

Figure S2. Morphology of *Chuandianella ovata* from the Chengjiang biota, Related to the STAR METHODS. (A) ELI SJZ-B14-860B, dorsal specimen showing detail information on carapace, antennules, eye and posterior tagma. Magnification of the rear of the posterior tagma (D) showing the anus, pseudo-segmented terminal ramus and lateral tail beams; (B) EJ-505, lateral specimen; (C) ELI SJZ-B21-779, a pair of multisegment antennules and eye extending beyond the anterior edge of carapace; (E) ELI SJZ-B20-909, sub-elliptical carapace with evenly pitted ornamentes. Abbreviations are as follows: S1–6: 1st to 6th segment.

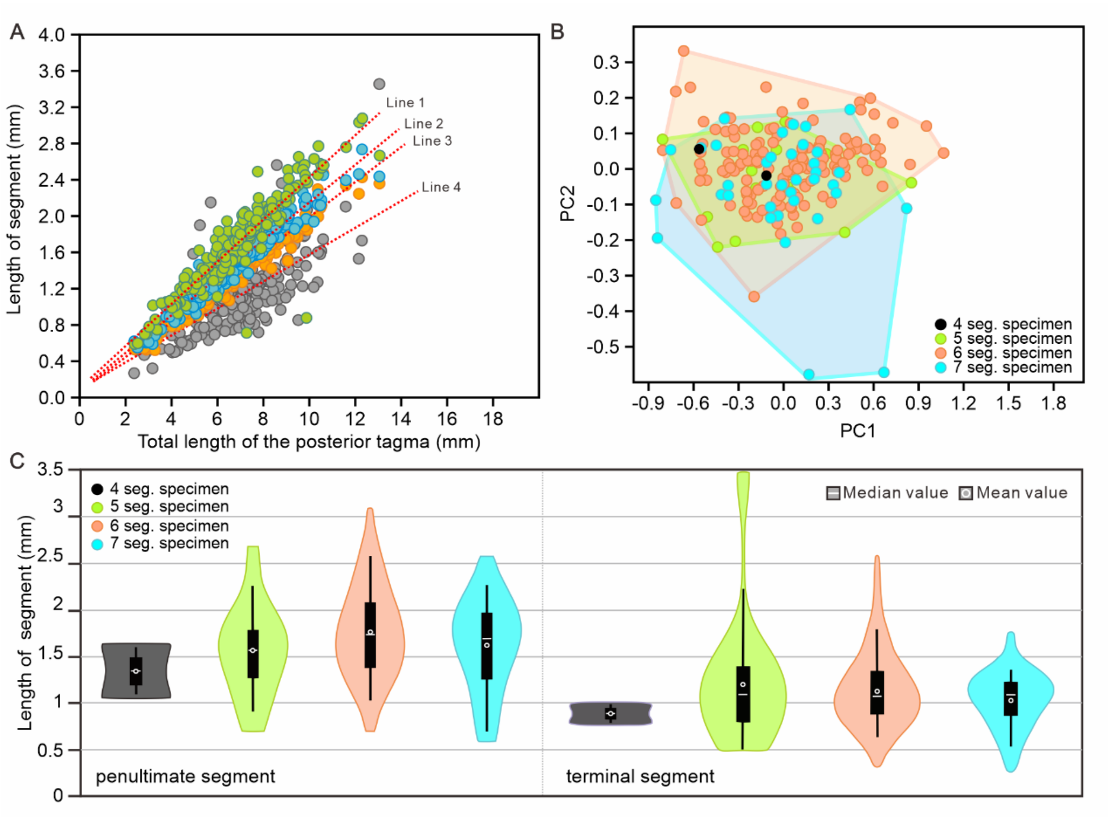

Figure S3. Statistical analysis of *Chuandianella ovata* posterior tagma, Related to the STAR METHODS. (A) The relationship between the length of the last four segments and the total length of posterior tagma. The fourth segment to last (orange points); the antepenultimate segment (blue points); the penultimate segment (green points); the terminal segment (gray points). Total length of the posterior tagma and each length of segment were referred to as independent (X) and dependent (Y) variables, respectively. Line 1 for the penultimate segment: Y=0.23228X+0.10558, R^2=0.85075; Line 2 for the antepenultimate segment: Y=0.20724X+0.077246, R^2=0.93559; Line 3 for the fourth segment to last: Y=0.19296X+0.060158, R^2=0.93246; Line 4 for the terminal segment: Y=0.14821X+0.092442, R^2=0.49731; (B) Principal components analysis of *C. ovata* in the length of posterior tagma, showing no variations between four categories; (C) Vioplot of the penultimate and

terminal segment among four categories of *C. ovata*.
